# The Influence of Saliva pH on the Fracture Resistance of Three Complete Denture Base Acrylic Resins

**DOI:** 10.1155/2020/8941876

**Published:** 2020-11-01

**Authors:** Juliana de Sá, Francisca Vieira, Carlos Manuel Aroso, Mónica Cardoso, José Manuel Mendes, António Sérgio Silva

**Affiliations:** ^1^Department of Oral Rehabilitation, Instituto Universitário de Ciências da Saúde, Gandra, Portugal; ^2^Dental Science Department, Instituto de Investigação e Formação Avançada em Ciências e Tecnologias da Saúde (IINFACTS), Gandra, Portugal

## Abstract

**Methods:**

Ten prosthesis bases prepared with each brand of resin were subjected to neutral and low pH conditions (pH 7 and pH 4) by submerging them in artificial saliva for 30 days. After exposure, the fatigue resistance of the resins was tested using a Dental CS® Testing Machine. *Statistical Analysis Test*. The data sets were described quantitatively in terms of mean (*M*) and standard deviation (SD). Shapiro–Wilk tests and unilateral analysis of variance (ANOVA) were performed and complemented by Tukey's multiple comparison tests. The effect size (*η*2), whose cohort points followed Cohen's recommendations: 0.01 (low), 0.06 (medium), and 0.14 (high), was calculated. The results were considered significant if *p* < 0.05 and marginally significant if *p* < 0.10.

**Results:**

One-way ANOVA showed that Megacryl® had the highest fracture resistance at pH 7 (52.23 Kgf), compared with Triplex Hot® (*p* < 0.001) and RS Vertex® (*p*=0.034). Two-way ANOVA confirmed the interaction between brand and pH (*p*=0.022), also revealing that brands comparison is significant or marginally significant, when pH is not considered (Megacryl® versus Triplex Hot®, *p* < 0.001, and RS Vertex®, *p*=0.058; Triplex Hot® versus RS Vertex®, *p*=0.051), and pH 7 results were significantly higher (*p*=0.003), even when brands are not considered. Hence, Megacryl® at pH 7 was found to have the highest fracture resistance, detached from other brands and pH values.

**Conclusion:**

It can be concluded within the limitations of this study that there are differences in the fracture resistance among the three brands of acrylic resin. Megacryl® was found to have the highest fracture resistance, and Triplex Hot® was the lowest. The results also show that exposure to a low pH environment decreases the fracture resistance of the Megacryl® and RS Vertex® resins.

## 1. Introduction

Oral rehabilitation aims to restore a patient's function and aesthetics by improving phonetics, nourishment, and facial aesthetics and, consequently, achieve better social integration and increased self-esteem. The percentages of all edentulous people have declined in several countries; however, with increasing average life expectancy and aging in the Western world, it is estimated that, by 2025, more than 50% of the population will be more than 50 years old. Despite advancements in oral hygiene, it is likely that many of these people will need full or partial dentures to replace missing teeth [1–3].

Acrylic resins, introduced by Wright in 1937, consist of polymethyl methacrylate (PMMA), which is formed by mixing a polymethyl methacrylate powder (polymer) and a methyl methacrylate liquid (monomer) (MMA). In acrylic resins, the correct powder-to-liquid ratio is important. They are typically mixed in a 3 : 1 ratio. There are three types of polymerization of acrylic resins: self-curing, heat-curing, and light-curing [4–7].

Heat-polymerizable resins have the following advantages: they are easy to process [4] and cost-effective and have excellent aesthetics [4, 8], low density, good repairability and relining [8], good dimensional stability [4, 8], good corrosion resistance, and low solubility in oral fluids. The disadvantages of acrylic resins include polymerization shrinkage, low tensile strength, low impact and fatigue resistance, high radiolucency, low thermal conductivity, and low flexibility (which permits fractures to occur). The low thermal conductivity of PMMA can lead to patient injury as a result of the loss of perception of cold and hot stimuli [3–12].

Due to their use in dentistry, acrylic resins based on PMMA must have the following characteristics: a natural appearance; high strength, rigidity, hardness, and dimensional stability; ease of handling; low density; accurate reproduction of surface details; absence of odor, taste, and toxic products; resistance to absorption of oral fluids; good retention of polymers, porcelain, and metals; resistance to bacterial growth; high radiopacity; ease of repair and cleaning; affordability; and good service life [3].

According to the American Dental Association, there should be no bubbles or voids in a denture base material when viewed without magnification. Porosity values above 11% have been linked to poor mechanical properties, impaired appearance, and retention of liquids and microorganisms. Porosity may weaken the prosthesis, resulting in high internal stress and making it more vulnerable to distortion and deformation. According to van Noort [3], approximately 30% of denture repairs performed in laboratories involve midline fracture, demonstrating that this is the most prevalent type of fracture in upper dentures [3].

The possibility of fracture due to low resistance to fatigue occurs because of the repetitive occurrence of stress in areas of the material with cracks that appear as a result of applied forces, especially chewing forces [5]. Fracture of a prosthesis due to impact is typically the result of some traumatic incidents, such as the prosthesis falling when the patient removes it from the mouth for cleaning. Although this may not necessarily result in an instant fracture, there is a possibility that a gap will form and continue to grow until the denture fractures. Inadequate strength of acrylic resin prostheses leads to fractures in 10% of prostheses during the first three years of use [3, 9].

Saliva, chewing, and diet can contribute to the degradation of acrylic resins. An immersed polymerized resin absorbs water because of the polar properties of the resin molecules, which lead to the diffusion of free monomers and other products. Salivary enzymes degrade polymers, resulting in the degradation of PMMA [5, 6, 10].

Because water absorption influences resin alteration, resin behavior within the oral cavity can be simulated using artificial saliva. The performance of materials in the oral cavity should be evaluated using artificial saliva of known composition because natural saliva varies greatly. It is important to note that it is impossible to obtain artificial saliva that precisely reproduces the characteristics of human saliva, which is very inconsistent and unstable [11, 13].

One reason for this instability is that the pH of human saliva varies in response to numerous factors throughout the day. Ingestion of certain foods can cause salivary pH to change from a value considered neutral to a lower value considered acidic. Fermentation of carbohydrates by bacteria in the oral cavity leads to a decrease in pH. Another condition that can trigger a decrease in oral pH levels is bulimia, in which gastric acid is eliminated through vomiting. Gastroesophageal reflux disease (GERD), one of the most prevalent digestive diseases today, can also lead to a decrease in oral pH, as reported by several studies [14–16].

As saliva plays such an essential role in oral homeostasis, changes in salivary flow must be considered. Hyposalivation is characterized by a marked reduction in salivary flow and is usually associated with xerostomia. This can have several causes, such as diabetes mellitus and Sjögren's syndrome. Drug use is another possible cause of this condition [17–19].

The acidity of the oral environment can have numerous consequences, such as increased susceptibility to dental caries, periodontal tissue disease, dysgeusia, halitosis, and an increased incidence of oral infections. Prosthetic stomatitis can occur in patients with dental prostheses because of fungal growth. Another consequence of a decrease in oral pH is the constant contact of dental prosthesis acrylic resins with acidic saliva. Studies showed that this acidity can lead to degradation of acrylic resins with resulting in decreased microhardness, greater release of residual monomer, and decreased fracture resistance. Therefore, dental prostheses subject to acidic pH levels can be expected to be more fragile and, therefore, are more prone to fracture [20–22].

This study aimed to evaluate the influence of oral pH on fracture resistance in prosthetic bases made with three different brands of thermopolymerizable acrylic resin and which of the three resins was the most resistant to fracture.

## 2. Materials and Methods

### 2.1. Materials

Heat-polymerizable acrylic resin plates (RS Vertex®, Triplex Hot®, and Megacryl®) (Table 1), artificial saliva (Fusayama–Meyer®), a Dental CS® test machine, and a Memmert® glasshouse were used.

### 2.2. Sample Preparation

**Figure 1:**
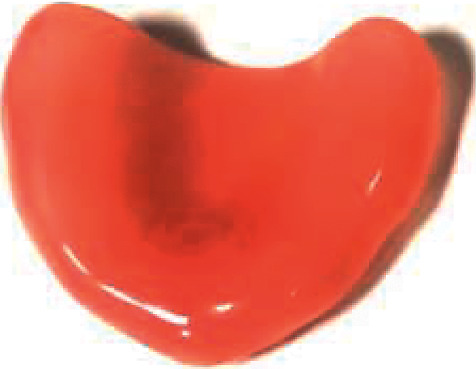
Example of an acrylic resins RS Vertex® sample.

**Figure 2:**
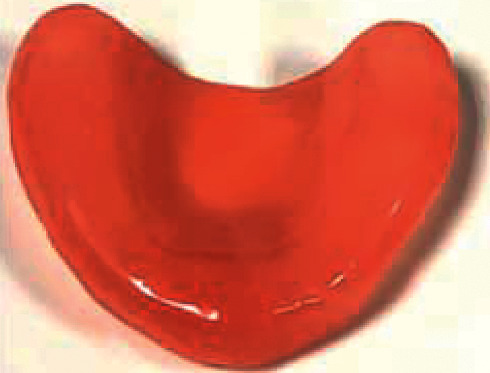
Example of an acrylic resins Triplex Hot® sample.

**Figure 3:**
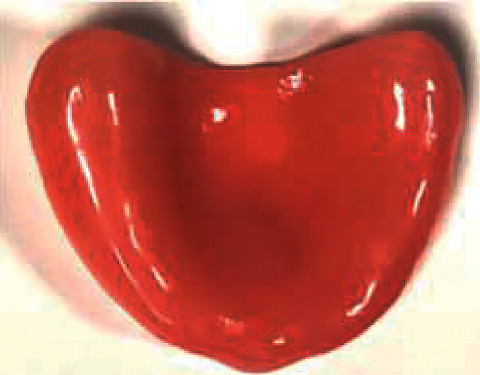
Example of an acrylic resins Megacryl® sample.

**Figure 4:**
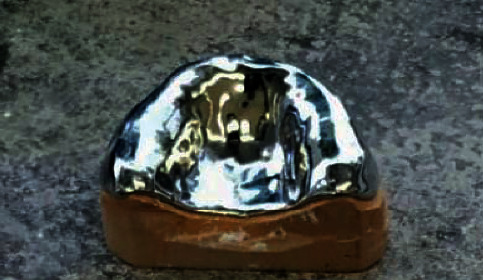
Prefabricated model.

**Figure 5:**
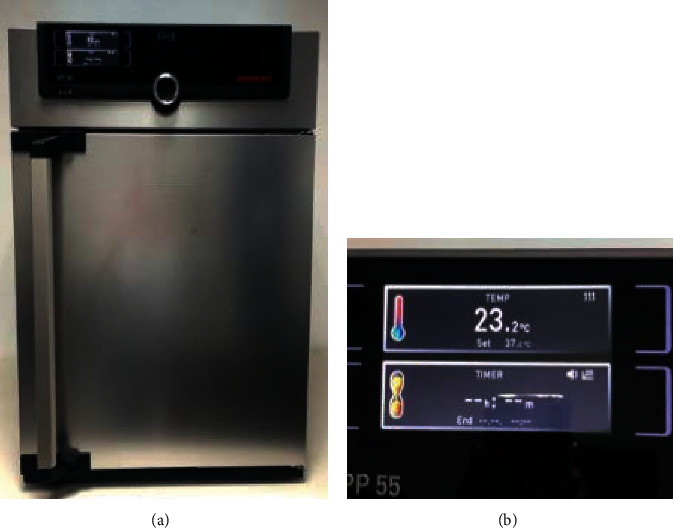
Memmert® glasshouse (a) at 37°C (b).

**Figure 6:**
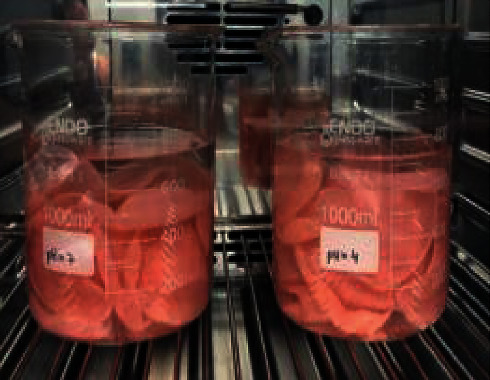
Prosthesis bases subjected to different pH values (pH 4 and pH 7).

**Figure 7:**
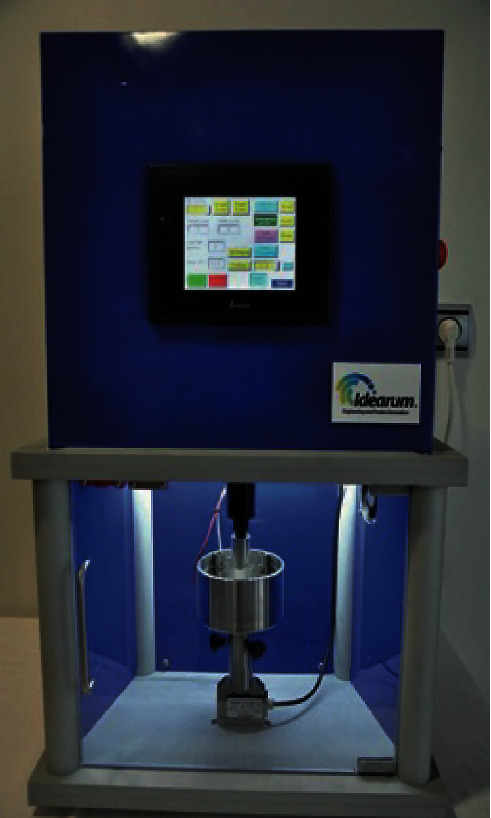
Dental CS® test machine.

The samples were prepared using three different brands of heat-polymerizable acrylic resins (RS Vertex®, Triplex Hot®, and Megacryl®). Ten prosthesis bases were formed using each brand, with dimensions of 60 × 45 mm^2^ and 2 mm of height (Figures 1–3), using a standardized prefabricated model (Figure 4). Fusayama–Mayer solution of artificial saliva (0.4 g/L NaCl, 0.4 g/L KCl, 0.795 g/L CaCl_2_.2H_2_O, 0.005 g/L Na_2_S.9H_2_O, 0.69 g/L NaH_2_PO_4_.2H_S_O, and 1 g/L urea) at 37 ± 2°C was used. Two different pH values (pH 4 and pH 7) were obtained by incorporating HCl in the base formula. Before starting the mechanical fatigue test, all the materials under the study were immersed in artificial saliva with the two different pH values to subject them to corrosion for 30 days. The incubator (Memmert, Germany) was heated to 37°C to simulate the conditions of oral temperature (Figures 5(a) and 5(b)), and the pH of the containers was measured 3 times per week for 30 days, using a digital pH meter (Ebro Electronic PTH 810, Germany). Fusayama–Mayer is well-known artificial saliva for testing products for corrosion, colorfastness, and discoloration and is used for testing a wide variety of products, including dental metal alloys. Considering a 3 × 2 design with 6 groups, *α* = 0.05, power = 0.80, and a maximum estimated effect size of *η*^2^ = 1.2, and the minimum sample size was 29. Hence, 5 samples were allocated to each group. Each base was submerged in 650 mL of artificial saliva with a pH of either 4 or 7 (Figure 6). After this exposure, the fatigue resistance of the acrylic resins was tested using a Dental CS® Testing Machine (Figure 7).

CS® Dental Testing Machine is a fatigue test device built in agreement with 2006/42/CE safety of machines and the norms EN 12100-1/2, EN 954-1, EN 1037, EN 61310-1/2, EN 60204-1, EN ISSO 14121-1, and EN ISSO 13850. The basic characteristics of the machine are a motor with 150 mm stroke, 1600 N maximum force, 210 mm/s speed, 1.06 mm/s speed of actuator, and 3000 rpm maximum speed forward. The cell of load had a precision of 0.01 N and a maximum force of 1500 N.

**Figure 8:**
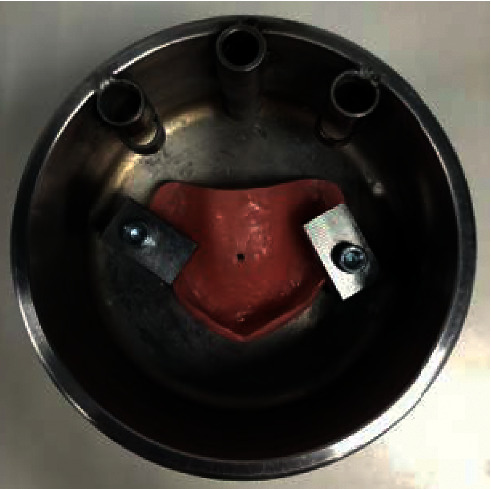
Prosthesis base placed to test the impact resistance. A point was marked at the center of the palate.

**Figure 9:**
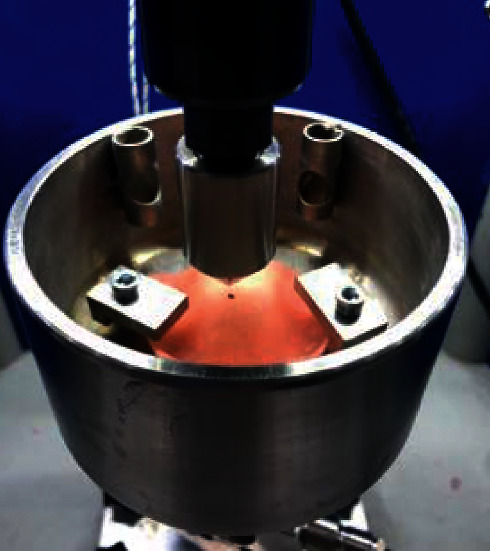
Peak dislocation toward prosthesis base.

**Figure 10:**
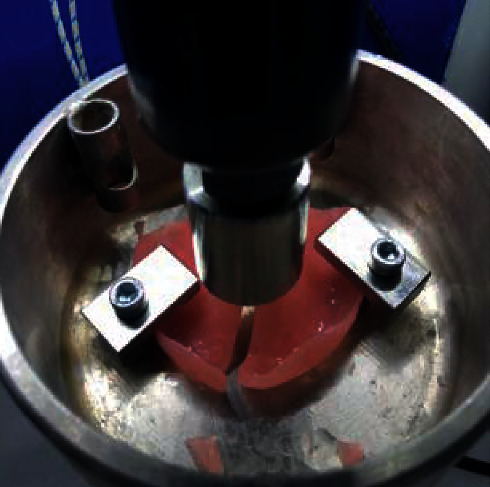
Prosthesis base fracture.

A point was marked at the center of the palate of each acrylic resin base (the fracture point was determined by taking the mean measure between the anteroposterior and mediolateral extremities of the denture), and the peak dislocation was measured at the point at which the acrylic fractured (Figures 8–10). The fracture force depended on the resistance of the acrylic and was detected by the load cell. A built-in program of the machine registers 4 points of strength per second. The maximum point of force, determined by the load cell where the rupture of the prosthesis occurs, was considered the fracture point. The test results were transferred to a Microsoft Office Excel® spreadsheet.

### 2.3. Statistical Analysis

The data were analyzed using SPSS version 22 (IBM Corporation, 2013). The data sets were described quantitatively in terms of mean (*M*) and standard deviation (SD). Shapiro–Wilk tests were conducted to assess the normality of the distributions of the force data for each resin type and pH level. The results confirmed that the assumption of normality was valid in all cases (*p* > 0.05). One-way analyses of variance (ANOVAs), complemented by Tukey's multiple comparison tests, were conducted to compare the average forces applied to the point of fracture for the three brands of acrylic resin at each pH level. The mean forces applied to fracture the resins at both pH levels were compared by means of two-way ANOVAs, complemented by Tukey's multiple comparison tests and calculation of the effect size (*η*^2^), whose cohort points followed Cohen's [1988] recommendations: 0.01 (low), 0.06 (medium), and 0.14 (high). The results were considered significant if *p* < 0.05  and marginally significant if *p* < 0.10.

### 2.4. Qualitative Analysis of Acrylic Plates

**Figure 11:**
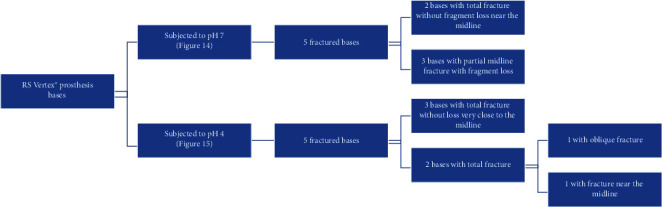
Observations concerning the fracture resistance performance of the acrylic RS Vertex® plates.

**Figure 12:**
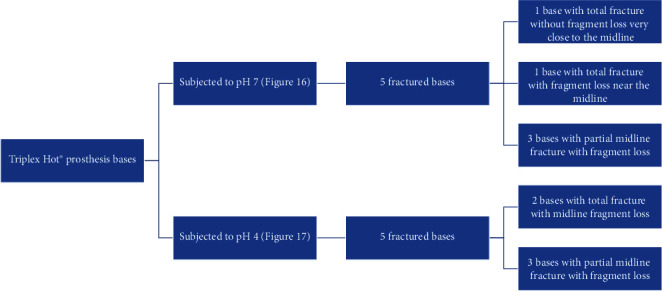
Observations concerning the fracture resistance performance of the acrylic Triplex Hot® plates.

**Figure 13:**
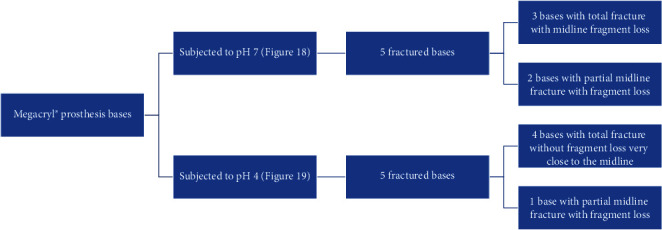
Observations concerning the fracture resistance performance of the acrylic Megacryl® plates.

Prior to testing, none of the plates exhibited notable porosity (Figures 11–13).

**Figure 14:**
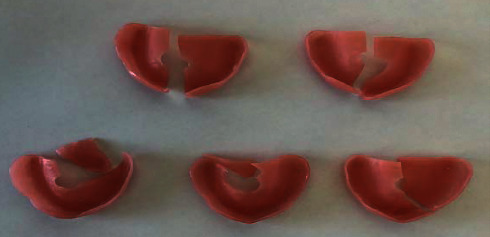
Megacryl® prosthesis bases subjected to pH 7 after fracture.

**Figure 15:**
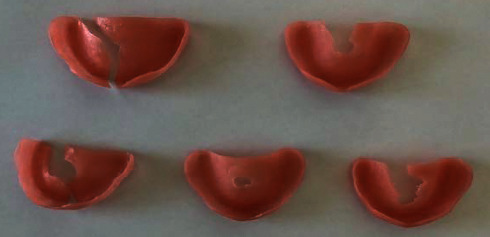
Triplex Hot® prosthesis bases subjected to pH 4 after fracture.

**Figure 16:**
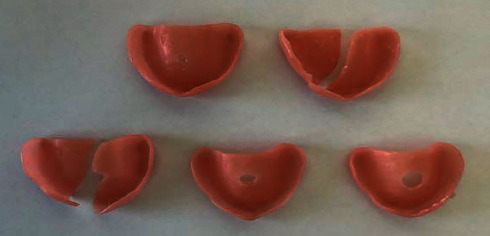
Triplex Hot® prosthesis bases subjected to pH 7 after fracture.

The Megacryl® subjected to pH 7 and the Triplex Hot® subjected to pH 4 exhibited the highest fragment loss during fracture (Figures 14 and 15, respectively) (Figure 16).

**Figure 17:**
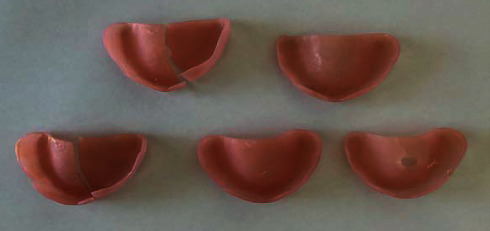
RS Vertex® prosthesis bases subjected to pH 7 after fracture.

**Figure 18:**
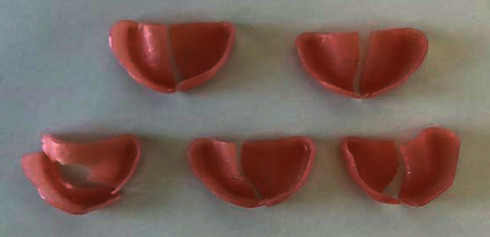
RS Vertex® prosthesis bases subjected to pH 4 after fracture.

At both pH levels, RS Vertex® exhibited the lowest incidence of fragment loss (five plates without total loss) (Figures 17 and 18, respectively).

More than half of the acrylic plates (17 of 30 plates) fractured fully very close to the midline zone, regardless of whether fragments were lost.

## 3. Results

### 3.1. Average Force Applied to Fracture

**Figure 19:**
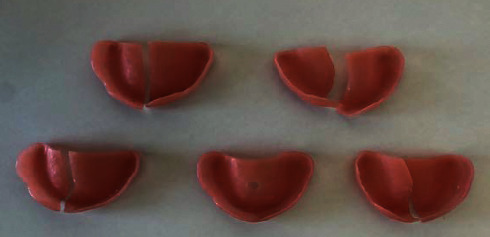
Megacryl® prosthesis bases subjected to pH 4 after fracture.

The Megacryl® resin had the highest fracture strength at both pH 7 (52.23 Kgf) and pH 4 (33.29 Kgf). RS Vertex® was the next, with strength values of 40.06 Kgf at pH 7 and 27.94 Kgf at pH 4. The Triplex Hot® brand had the lowest fracture strength at both pH 7 (23.87 Kgf) and pH 4 (26.15 Kgf) (Table 2) (Figure 19).

### 3.2. Time to Fracture

**Figure 20:**
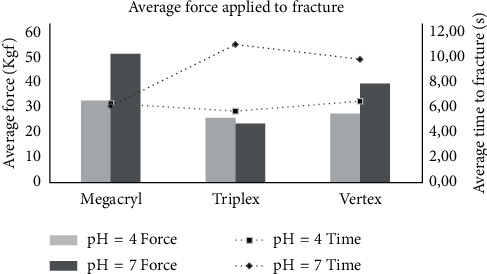
Time and average strength applied until fracture in three different acrylic resin brands by pH.

On average, bases subjected to pH 7 took longer to fracture, particularly for Triplex Hot® and RS Vertex®. For Megacryl®, the times to fracture were very similar for the two pH levels (Figure 20).

### 3.3. Comparison of the Average Force Applied to Fracture in the Three Brands of Acrylic Resin Separated by Salivary pH Type Used

For the pH 4 environment, no statistically significant differences were detected for the three considered brands: *F*_(2.12)_ = 1.33 (*p*=0.301).

For the pH 7 environment, significant differences between the brands were detected: *F*_(2.12)_ = 12.86 (*p* < 0.001). This was particularly true between the Triplex Hot® and Megacryl® brands (*p* < 0.001) and between the Triplex Hot® and Vertex RS® brands (*p*=034) (Table 3).

### 3.4. Comparison of the Average Force Applied until Fracture, considering the Interaction between the Brand and the pH

Tables 4–6 present the results of the two-way ANOVAs conducted to compare the mean fracture force by brand and pH and by their interaction.

The overall results (Table 4) show that the average force applied to fracture varies by brand, *F*_(2.24)_ = 12.07 (*p* < 0.001), pH, *F*_(1.24)_ = 10.58 (*p*=0.003), and the interaction brand × pH, *F*_(2.24)_ = 4.50 (*p*=0.022).

Table 5 shows that, regardless of pH, there were statistically significant differences between Megacryl® and Triplex Hot® (*p* < 0.001) and marginally significant differences between the latter and RS Vertex® (*p*=0.051). Marginally significant differences were also observed between Megacryl® and the RS Vertex® (*p*=0.058).

Tukey's multiple comparison tests also revealed statistically relevant differences between pH levels, regardless of brand (*p*=0.003) (Table 6).

## 4. Discussion

The study of dental materials involves observation of their characteristics and assessment of whether they benefit patients. For acrylic resin used in dental prostheses, one of the most important aspects of its performance is its fracture resistance. This study was conducted to evaluate which of three brands of the acrylic resin provides the greatest fracture resistance. Prostheses are known to experience occasional failures such as midline fractures in full dentures, tooth detachment, and other types of total or partial denture failure. Research into the causes of repair involving full and partial dentures has shown that 30% of all repairs are associated with midline fractures. Acrylic prostheses can still fracture due to fatigue or shock caused by chewing, making it difficult to repair the acrylic resin. Chewing is a repetitive force that results in generalized cracks that weaken the base of the prosthesis, ultimately resulting in fracture. These types of fractures are often caused by patients who accidentally drop the prosthesis during their daily oral hygiene [3, 23, 24]. In this study, a point was marked in the central zone of each of the tested prosthesis bases to simulate this type of midline fracture.

The acrylic resin brand Megacryl® was found to have the greatest fracture strength at both pH 7 (52.23 Kgf) and pH 4 (33.29 Kgf). The RS Vertex® brand exhibited the next highest fracture resistance values: 40.06 Kgf at pH 7 and 27.94 Kgf at pH 4. The Triplex Hot® brand exhibited the lowest fractures strengths at pH 7 (23.87 Kgf) and pH 4 (26.15 Kgf).

At pH 7, there were significant differences between the Triplex Hot® and the two competitors (Megacryl® and RS Vertex®). At pH 4, the mean strength values up to fracture were similar for the three brands.

Another objective of this study was to assess whether oral pH influenced the fracture resistance of acrylic resins. It was concluded that, after exposure to an environment of pH 7, a higher average force to fracture could be sustained. A significant interaction between the brand and the pH was also detected, with a higher fracture resistance being exhibited by the Megacryl® and RS Vertex® brands after pH 7 exposure, but the same fracture as the Triplex Hot® brand, for which the results for pH 4 and pH 7, was very similar.

These results suggest that acidic conditions (pH 4) may lead to decreased fracture resistance, as demonstrated by the results for the brands Megacryl® and RS Vertex®. This was consistent with the results reported by Nicodemo et al. [22], who found that a low pH reduces the strength of acrylic resin, regardless of the processing technique. Their study demonstrated a reduction in microhardness after storage of samples in heptane and ethanol solutions with different concentrations selected to simulate the human diet. In another study, Tuna et al. demonstrated that acidic saliva conditions lead to a higher residual monomer release than neutral saliva conditions, decreasing the strength of acrylic resin.

These results reflect what happens daily with patients' acrylic prostheses because there are numerous reasons for lowered oral pH, such as sugars consumed in the diet, which result in bacteria in the oral cavity that produce acid [14, 19].

Another possible cause is gastroesophageal reflux, in which there is a spontaneous movement of gastric contents from the stomach to the esophagus and a consequent reduction in salivary pH. It has also been reported that patients with disease and/or drug-induced xerostomia and hyposalivation are more likely to have a more acidic oral pH because the buffering effect of saliva is diminished when it is produced in reduced amounts. This results in an increase in the number of microorganisms present in the oral cavity, which makes the medium become more acidic [25].

With respect to the time to fracture, samples of Triplex Hot® and RS Vertex® subjected to a pH 4 failed sooner than samples of Megacryl®, for which the time to fracture was the same for both pH values. These results reflect the decreased strength of acrylic resin under acidic pH conditions and, therefore, the sooner occurrence of fractures.

Overall, Megacryl® acrylic resin was the toughest and, therefore, required the greatest strength to fracture at complete fragmentation. The other two brands of acrylic resin fractured in a more homogeneous and linear manner.

This work has as main clinical objective to determine the basic characteristics of the main acrylics used in rehabilitation, to find the best material for a given rehabilitation and clinical condition of the patient.

The limitations of this study are due to the fact that it is in vitro; despite trying to mimic the oral cavity and its conditions as much as possible, there are always differences. In future investigations, it will be important to perform a long-term follow-up in vivo study.

## 5. Conclusions

Based on the results obtained using the methods and materials described in this study, the following conclusions can be drawn.

There are differences in fracture resistance among the three brands of heat-polymerizable acrylic resin analyzed in this study. The brand Megacryl® exhibited the highest fracture resistance, followed by RS Vertex® and Triplex Hot®.

A low pH environment reduces the fracture resistance of the Megacryl® and RS Vertex® acrylic resins. The Triplex Hot® brand did not exhibit an effect of pH on fracture.

## Figures and Tables

**Table 1:** Acrylic resin characteristics.

Brand	Compositions	Proportion	Polymerization techniques
RS Vertex® Vertex-Dental B.V.	Powder: polymethyl methacrylate, catalyst, pigments Liquid: methyl methacrylate, dimethacrylate	1 mL/0.95 g liquid (monomer) 2.3 g powder (polymer)	20 min at 100°C
Triplex Hot® Ivoclar Vivadent	Powder: polymethyl methacrylate, catalyst, pigments Liquid: methyl methacrylate stab, dimethacrylate	1 mL liquid (monomer) 2.34 g powder (polymer)	45 min at 100°C
Megacryl Hot® Megadental GmbH	Powder: polymethyl methacrylate, catalyst, pigments Liquid: methyl methacrylate, dimethacrylate	4 g liquid (monomer) 10 g powder (polymer)	25–30 min at 98–100°C

**Table 2:** Average force (Kgf) applied to fracture of the three brands of acrylic resin by pH.

Brand	pH = 4 (*n* = 15)	pH = 7 (*n* = 15)
Megacryl® (*n* = 10)	33.29 (10.89)	52.23 (12.44)
Triplex Hot® (*n* = 10)	26.15 (3.35)	23.87 (7.91)
RS Vertex® (*n* = 10)	27.94 (5.04)	40.06 (4.34)

Results are presented in *M (DP)* format.

**Table 3:** One-way ANOVA test for comparison of the three pH-separated acrylic resin brands.

Tukey tests					
pH	*F* test	*p* value	Megacryl® versus Triplex Hot®	Megacryl® versus RS Vertex®	RS Vertex® versus Triplex Hot®
pH = 4	*F*_(2.12)_ = 1.33	*p*=0.301	*p*=0.296	*p*=0.489	*p*=0.919
pH = 7	*F*_(2.12)_ = 12.86	*p* < 0.001^*∗∗∗*^	*p* < 0.001^*∗∗∗*^	*p*=0.117	*p*=0.034^*∗*^

^*∗*^ *p* < 0.05;^*∗∗*^*p* < 0.01; ^*∗∗∗*^*p* < 0.001.

**Table 4:** Global tests for brand effect, pH, and brand × pH interaction.

Brand	*F* test	*p* value	Effect size (*η*^2^)
Brand	*F*_(2.24)_ = 12.07	*p* < 0.0001^*∗∗∗*^	*η* ^2^ = 0.50
pH	*F*_(1.24)_ = 10.58	*p*=0.003^*∗∗*^	*η* ^2^ = 0.31
Brand × pH	*F*_(2.24)_ = 4.50	*p*=0.022^*∗*^	*η* ^2^ = 0.27

^*∗*^ *p* < 0.05;^*∗∗*^*p* < 0.01;^*∗∗∗*^*p* < 0.001.

**Table 5:** Tukey multiple global comparisons between brands.

Brand	Megacryl®	Triplex Hot®	RS Vertex
Megacryl®	—		
Triplex Hot®	*p* < 0.001^*∗*^	—	
RS Vertex®	*p*=0.058^†^	*p*=0.051^†^	—

^*∗*^ *p* < 0.001;^†^*p* < 0.10.

**Table 6:** Tukey multiple global comparisons between pH valuess.

Comparisons	*p* value
pH 4 versus pH 7	*P*=0.003^*∗∗*^

^*∗∗*^ *p* < 0.01.

## Data Availability

The data used to support the findings of this study are available from the corresponding author upon request.
